# Transcriptome profiling of fast/glycolytic and slow/oxidative muscle fibers in aging and obesity

**DOI:** 10.1038/s41419-024-06851-y

**Published:** 2024-06-28

**Authors:** Feng-Min Zhang, Hao-Fan Wu, Ke-Fan Wang, Ding-Ye Yu, Xian-Zhong Zhang, Qi Ren, Wei-Zhe Chen, Feng Lin, Zhen Yu, Cheng-Le Zhuang

**Affiliations:** 1 Department of Gastrointestinal Surgery, Shanghai Tenth People’s Hospital, Tongji University School of Medicine, Shanghai, China; 2 Department of Orthopaedic Surgery, Sir Run Run Shaw Hospital, Zhejiang University School of Medicine, Hangzhou, China; 3 Key Laboratory of Musculoskeletal System Degeneration and Regeneration Translational Research of Zhejiang Province, Hangzhou, China; 4 Department of General Surgery, Huadong Hospital, Fudan University, Shanghai, China

**Keywords:** Ageing, Fat metabolism

## Abstract

Aging and obesity pose significant threats to public health and are major contributors to muscle atrophy. The trends in muscle fiber types under these conditions and the transcriptional differences between different muscle fiber types remain unclear. Here, we demonstrate distinct responses of fast/glycolytic fibers and slow/oxidative fibers to aging and obesity. We found that in muscles dominated by oxidative fibers, the proportion of oxidative fibers remains unchanged during aging and obesity. However, in muscles dominated by glycolytic fibers, despite the low content of oxidative fibers, a significant decrease in proportion of oxidative fibers was observed. Consistently, our study uncovered that during aging and obesity, fast/glycolytic fibers specifically increased the expression of genes associated with muscle atrophy and inflammation, including Dkk3, Ccl8, Cxcl10, Cxcl13, Fbxo32, Depp1, and Chac1, while slow/oxidative fibers exhibit elevated expression of antioxidant protein Nqo-1 and downregulation of Tfrc. Additionally, we noted substantial differences in the expression of calcium-related signaling pathways between fast/glycolytic fibers and slow/oxidative fibers in response to aging and obesity. Treatment with a calcium channel inhibitor thapsigargin significantly increased the abundance of oxidative fibers. Our study provides additional evidence to support the transcriptomic differences in muscle fiber types under pathophysiological conditions, thereby establishing a theoretical basis for modulating muscle fiber types in disease treatment.

## Introduction

Skeletal muscle is composed of muscle cells, also known as muscle fibers due to their long and fibrous shape. Muscle fibers are heterogeneous and are broadly divided into two major categories as fast/glycolytic fibers and slow/oxidative fibers, whose differences involve physiological distribution, contractile properties, metabolic pathway, and all functional cell compartments [1]. Recent researches highlight the importance of these fibers. Through muscle-specific knockouts of DP2 or Sox6, enhancements in endurance and insulin sensitivity are achieved by increasing the number of slow/oxidative fibers [2, 3]. Additionally, constitutive activation of Akt elevates fast/glycolytic fibers, promoting muscle hypertrophy and strength gains [4]. However, the majority of muscles are made up of a mixture of both slow- and fast-twitch fibers in vivo, posing challenges for research focusing on specific fiber types. Soleus (SOL) and extensor digitorum longus (EDL) are two widely recognized natural slow/oxidative and fast/glycolytic fibers, offering a foundation to explore biological differences in skeletal muscle fiber types.

Aging and obesity are prominent contributors to muscle atrophy, characterized by the reduction in muscle mass due to the aging process and the accumulation of fat [5, 6]. The essence of muscle atrophy lies in the atrophy of specific muscle fiber types. During aging, fast-twitch fibers are vulnerable, as evidenced by more pronounced atrophy [7, 8]. Therefore, the conventional perspective believes that aging is associated with a decrease in fast-twitch fibers and an increase in slow-twitch fibers [9]. However, contradictory findings exist, suggesting that slow-twitch fibers may also decrease and exhibit marked atrophy behavior in advanced age [10–13]. In the case of obesity, evidence indicates that obese individuals had increased fast-twitch fibers [14]. However, the distribution of different fiber types exhibits spatial specificity, but most studies analyze local areas rather than the entire cross-section of fibers [15–18]. Currently, it remains unclear whether the changes in fiber types are consistent under different physiological and pathological conditions, and the trend of these changes is also unclear.

Here, we observed a significant reduction in the proportion of slow/oxidative fibers and an increasing trend in fast/glycolytic fibers in the tibialis anterior (TA), regardless of aging or obese status. Additionally, we performed RNA-seq analysis on the EDL and SOL, and identified a significant a number of differentially expressed genes (DEGs) in response to aging and obesity. Our study uncovered that fast/glycolytic fibers tend to upregulated pro-atrophy genes, while slow/oxidative fibers tend to express genes associated with cellular protection during aging and obesity. Notably, our analysis indicated the potential central role of calcium-related signaling pathways in these processes. Treatment with the sarco/endoplasmic reticulum Ca^2+^-ATPases (SERCA) inhibitor thapsigargin resulted in a significant increase in the proportion of oxidative fibers in mice. Our findings provide novel insights into the mechanisms of muscle fiber type changes and suggest a potential treatment strategy.

## Materials and methods

### Animals

Male were obtained from GemPharmatech Co., Ltd. (Nanjing, China). The young mice were aged 10 weeks, and the aged mice were 27 months, aligning with previous aging research [19, 20]. Diet-induced obesity (DIO) mice were fed a 60% high-fat diet at 6 weeks old, and last for 18 weeks. Animals were maintained in a specific pathogen-free environment on a 12/12 h light–dark cycle and fed rodent normal chow diet ad libitum. After animals were euthanized, TA, quadriceps, EDL, and SOL were harvested for analysis. Ethics approval was granted by Institutional Animal Committee of Tongji University. Mice used for this study received care throughout the experiment following the Guide for the Care and Use of Laboratory Animals.

### Assessment of grip strength

A grip strength meter (ZhongShi Biological Technology Co. Ltd., China) was used to measure the maximum grip strength. Mice were placed on the device, ensuring all paws touched the grid, and pulled horizontally until they released their grip. Five trials were conducted for each mouse.

### Glucose tolerance test

Blood glucose was measured using a portable blood glucose meter (Jiangsu Yuyue Medical Equipment & Supply Co., Ltd.) using mouse tail vein blood. The glucose tolerance test (GTT) was performed after an overnight fast by intraperitoneal injection of 2 g/kg glucose. Blood glucose levels were measured at 0, 15, 30, 60, 90, and 120 minutes after injection.

### Transcriptomic analysis

Total RNA was extracted from EDL and SOL using TRIzol reagent (Invitrogen Life Technologies, USA). RNA quality, concentration, and purity were assessed using a Nanodrop 2000 instrument (Thermo Fisher Scientific, USA). Libraries for next-gen sequencing were prepared with the TruSeqTM RNA Sample Prep Kit (Illumina, USA). Sequencing was performed by Shanghai Origingene Biopharm Technology Co., Ltd. (Shanghai, China). High-quality data were obtained after initial quality control and alignment to the reference genome using HISAT2 [21]. Expression values were calculated using the StringTie tool, and the differentially expressed genes (fold change≥2, adjusted *p*-value ≤ 0.05) were identified using the DESeq2 algorithm [22, 23]. GO annotation and KEGG pathway enrichment analysis were performed based on DAVID online database [24]. Gene Set Enrichment Analysis (GSEA) software (version 4.3.2) was used to perform gene set enrichment analysis [25]. Raw sequencing data are available at the NCBI SRA database (PRJNA1009685 and PRJNA1108514).

### Muscle histology and immunohistochemistry

Muscles were frozen in OCT (Epredia, #11912365), stored at −80 °C, and sectioned into 10 μm slices for subsequent analysis. Hematoxylin and eosin (H&E) staining were performed on the cryopreserved muscle sections. Immunofluorescence staining was conducted to assess myosin heavy chain (MHC) isoform expression with specific primary antibodies: anti-myosin I (DSHB, #BA-D5), anti-myosin IIA (DSHB, #SC-71), and anti-myosin IIB (DSHB, #BF-F3). Additionally, the fiber basal membrane was visualized using an anti-laminin antibody (Abcam, #ab11575). Alexa-488 or Alexa 594-labeled anti-mouse or anti-rabbit secondary antibodies were applied, and DAPI (Vector Laboratories, #H-1200) was used for nuclear staining. Digital images were acquired using the Olympus VS120 slide scanning system.

### Succinic acid dehydrogenase staining

Cryosections of 10 μm thickness were obtained from the muscle samples and were incubated with succinic acid dehydrogenase (SDH) stain kit (Solarbio, #G2000) at 37 °C for 20 min. After washed with PBS, the slides are sealed with glycerine gelatin. Digital images were acquired using the Olympus VS120 slide scanning system.

### Pharmacological treatments

Mice were injected with thapsigargin or normal saline intraperitoneally (i.p.) every day for 1 month. Thapsigargin was solubilized in normal saline with finial concentration of 3 mg/kg for injection.

### Statistical analysis

Statistical analysis was performed using Graph Pad Prism 9.0 software. All data are expressed as means ± SEM. Student’s t-test was used for statistical analysis between two groups, with either one-tailed or two-tailed tests as appropriate. For experiments involving more than two groups, one-way analysis of variance (ANOVA) followed by post hoc tests was applied to assess the variation among groups. Differences between groups were considered statistically significant for *P* < 0.05. All experiments were independently repeated three times.

## Results

### Aging and obesity lead to significant muscle atrophy

To investigate the effects of aging and obesity on skeletal muscle, we established three groups: a control group of 10-week-old wild-type mice, an aged group of 27-month-old mice, and an obese group of 10-week-old Ob/Ob mice. Aged and Ob/Ob mice had significantly higher body weight and low relative grip strength (Supplementary Fig. 1A, B). Besides, Ob/Ob mice exhibited significant glucose intolerance, while the glucose tolerance of aging mice was comparable to that of the control group (Supplementary Fig. 1C).

**Fig. 1:**
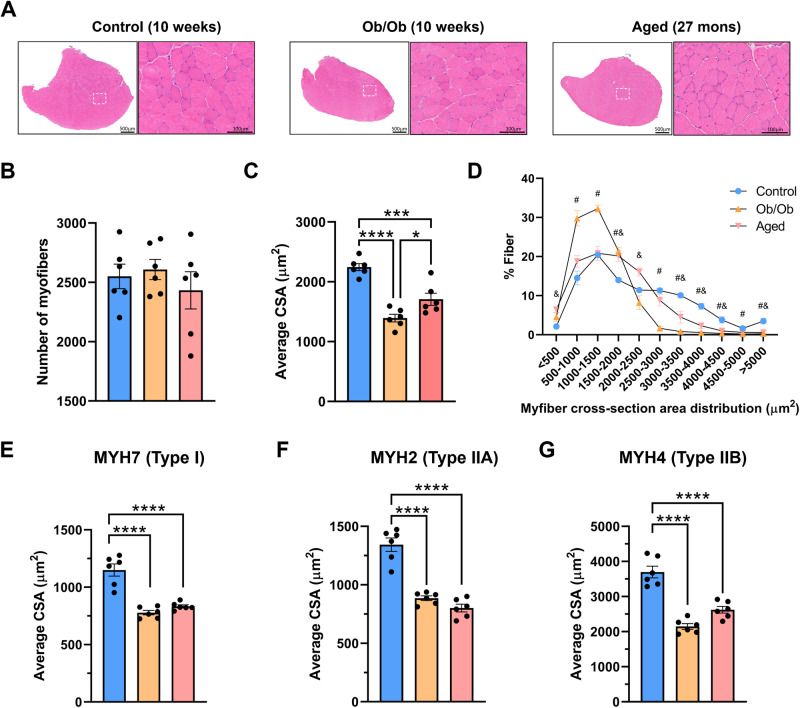
Aging and obesity result in a significant decline in muscle mass. **A** Hematoxylin and eosin (H&E) staining of tibialis anterior (TA) sections. **B** Statistical results of the number of muscle fibers. **C** Statistical results of the average cross-sectional area (CSA) of TA fibers. **D** Distribution of muscle fiber CSA, data are expressed as percentages. **E** Average CSA of type I fibers. **F** Average CSA of type IIA fibers. **G** Average CSA of type IIB fibers. *n* = 3–6; ^*^P < 0.05, ^**^P < 0.01, ^***^P < 0.001, ^****^P < 0.0001; ^#^ indicated control group vs. Ob/Ob group, *P* < 0.05; ^&^ indicated control group vs. aged group, *P* < 0.05.

To explore the effects of aging and obesity on muscle fibers, mice were sacrificed to collect their TA for assessment of atrophy by H&E staining (Fig. 1A). The results revealed that both aging and obesity significantly reduced the cross-sectional area (CSA) of muscle fibers in the TA, without affecting their quantity (Fig. 1B–D). All three types of fibers experience atrophy during the aging and obesity (Fig. 1E–G), but the greater size of type IIB fibers contribute more to overall muscle atrophy. Together, these data show that aging and obesity lead to significant skeletal muscle atrophy.

### Aging and obesity lead to a reduction in slow/oxidative fibers and an increase in fast/glycolytic fibers in mixed muscles

**Fig. 2:**
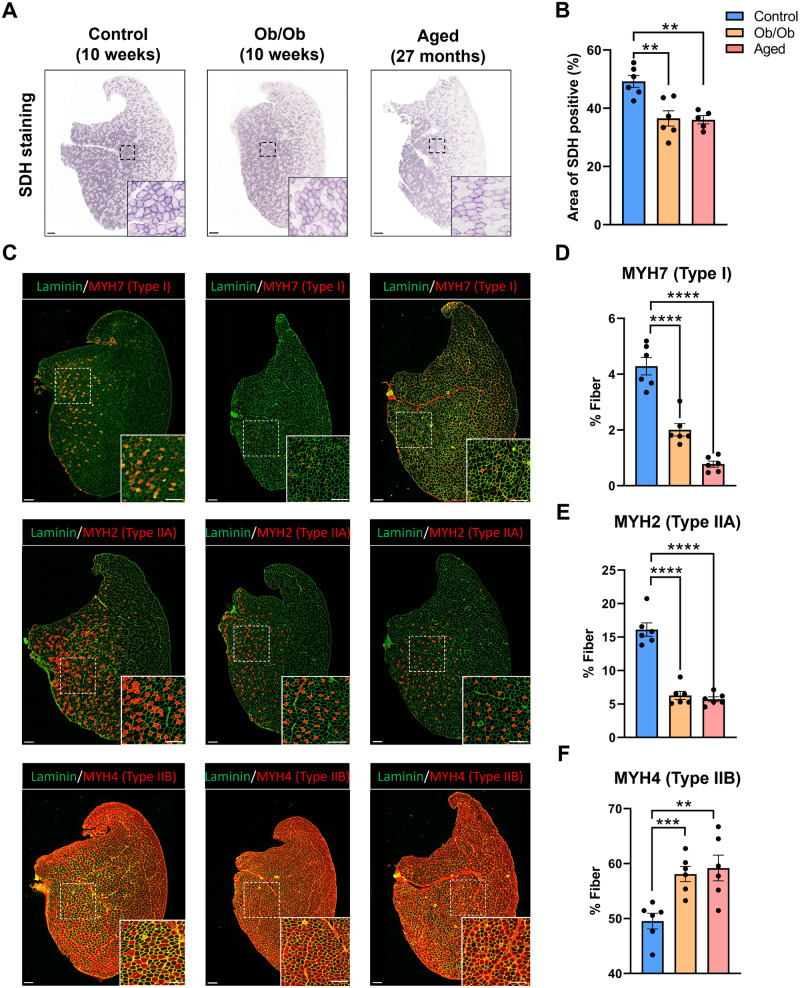
Aging and obesity lead to a decrease in the proportion of oxidative fibers in TA. **A** Succinate dehydrogenase (SDH) staining of TA sections in control, aged, and Ob/Ob mice, Scale bar: 200 μm. **B** Statistical results of the percentage of the SDH positive area to total area. **C** Immunofluorescence staining of MYH7, MYH2, MYH4 of TA sections. Scale bar: 200 μm. **D** Statistical results of the proportion of type I fibers. **E** Statistical results of the proportion of type IIA fibers. **F** Statistical results of the proportion of type IIB fibers. Data are expressed as percentages; *n* = 5–6; ^**^P < 0.01, ^***^P < 0.001, ^****^P < 0.0001.

Previous studies usually analyze fiber types based on local area. In this study, we conducted our analysis on the entire muscle cross-sections. Aging and obesity had significantly decreased SDH positive area in TA muscle (Fig. 2A, B), suggesting a decline in mitochondrial function. During aging and obesity, the proportions of type I and IIA fibers were significantly decreased, whereas the proportion of type IIB fibers were increased in TA (Fig. 2C–F). Type IIA represents a distinct fast-twitch fiber subtype, characterized by a metabolic profile resembling that of type I [1]. Consistent results were obtained for immunofluorescent staining of type I and type IIA fibers in another mixed muscle, quadriceps (Supplementary Fig. 2).

### Soleus and extensor digitorum longus muscles exhibit distinct changes in muscle fiber types during the aging and obesity processes

**Fig. 3:**
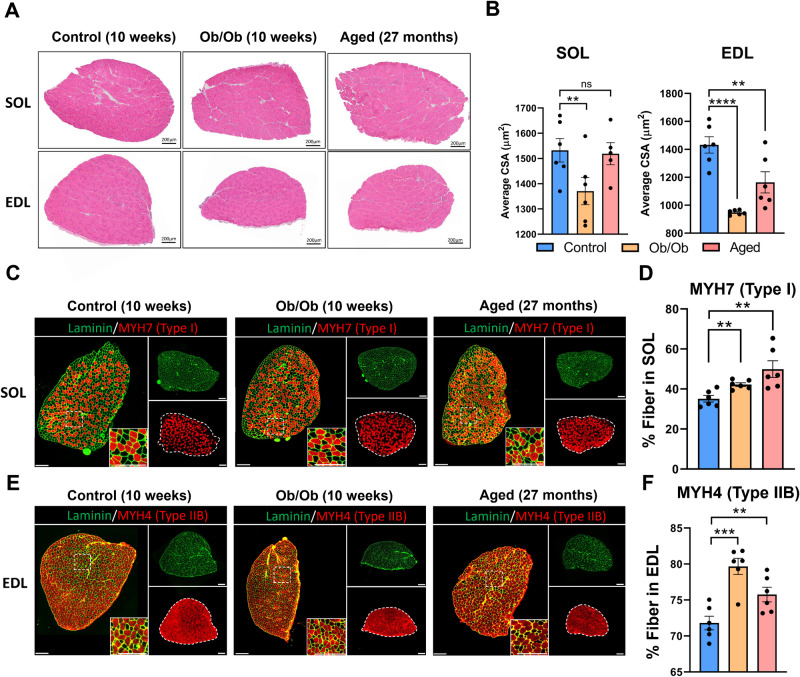
SOL and EDL exhibit different changes in muscle fiber types composition during the aging and obesity processes. **A** H&E staining of SOL and EDL in control, aged, and Ob/Ob mice. **B** Statistical results of the CSA of SOL and EDL fibers. **C** Immunofluorescence staining of MYH7 for SOL, Scale bar: 200 μm. **D** Statistical results of the proportion of type I fibers in SOL. **E** Immunofluorescence staining of MYH4 for EDL, Scale bar: 200 μm. **F** Statistical results of the proportion of type IIB fibers in EDL. *n* = 5–6; ^**^P < 0.01, ^***^P < 0.001, ^****^P < 0.0001.

To investigate the effects of aging and obesity on muscles with higher proportion of slow- or fast-twitch fibers, we performed H&E, SDH, and immunofluorescent staining on soleus (SOL) and extensor digitorum longus (EDL) from control, aged, and Ob/Ob group. Most of the SOL primarily comprises oxidative fibers, in contrast to the EDL, which has minimal oxidative fiber content (Supplementary Fig. 3A). Among them, SOL is primarily composed of type I (~40%) and type IIA (~50%), while EDL is predominantly composed of type IIB (~70%; Fig. 3C–F and Supplementary Fig. 3B, C). In the SOL, CSA significantly decreased in Ob/Ob mice, while it remained unchanged in the aged group. In the EDL, both the aged and obese EDL exhibited a decrease in CSA (Fig. 3A, B). During aging and obesity, the proportion of type I fibers in SOL not only did not decrease but showed a slight increasing trend (Fig. 3C, D). Despite the rarity of type I fibers in EDL, their proportion reduced significantly with aging and obesity, as type IIB fibers increased (Supplementary Fig. 3C and Fig. 3E, F).

### Characterizing inherent properties of fast/glycolytic and slow/oxidative fiber using RNA-seq

Considering the distinct trends of fiber type changes in SOL and EDL during aging and obesity, RNA-seq analysis was conducted on SOL and EDL samples from the three mouse groups (Supplementary Fig. 4A). Principal component analysis (PCA) and sample correlation matrix showed a strong clustering by samples and a good agreement between mice (Supplementary Fig. 4B, C), although the distinction between obese and control groups is less prominent. This may be attributed to the similar ages of the mice in the obese and control groups. Consistent with the immunofluorescent staining results, SOL predominantly expresses the marker genes MYH7 and MYH2 for type I and type IIA, while EDL mainly expresses the marker gene MYH4 for type IIB (Supplementary Fig. 4D–F).

To investigate the transcriptional differences between fast/glycolytic and slow/oxidative fibers, we analyzed sequencing data from SOL and EDL in 10-week-old control mice. The analysis identified 1155 protein-coding differentially expressed genes (DEGs), comprising 594 upregulated and 561 downregulated genes (FDR ≤ 0.05, log_2_FC ≥ 1). Four hundred and forty-two DEGs with an average FPKM > 10 in EDL or SOL were shown in the Supplementary File 1. Among them, top 10 upregulated and 10 downregulated genes were shown in Supplementary Table 1. Gene Oncology (GO), Kyoto Encyclopedia of Genes and Genomes (KEGG), and Gene Set Enrichment Analysis (GSEA) analysis were performed (Supplementary Fig. 5A–F). Fast/glycolytic fibers predominantly employ anaerobic glycolysis with enhanced sarcoplasmic reticulum and calcium ion transport. Genes governing glycolysis enzymes are upregulated in fast/glycolytic fibers (Supplementary Fig. 5G), in line with prior research [1]. Genes encoding key enzymes involved in aerobic metabolism are upregulated in slow/oxidative fibers (Supplementary Fig. 5H).

To further validate the stably existing DEGs between fast/glycolytic and slow/oxidative fibers, we intersected the DEGs across young, aging, and Ob/Ob mice (Supplementary Fig. 6A). We discovered 677 protein-coding DEGs consistently present in all three groups (Supplementary File 2). Notably, each DEG maintained a consistent up or downregulation across all groups, underscoring their muscle fiber specificity and independence from physiological changes. GO and KEGG analyses of these 677 DEGs yielded results in line with entire DEGs analysis in control mice (Supplementary Fig. 6B–F).

### Transcriptome changes of fast/glycolytic and slow/oxidative fibers during obesity

**Fig. 4:**
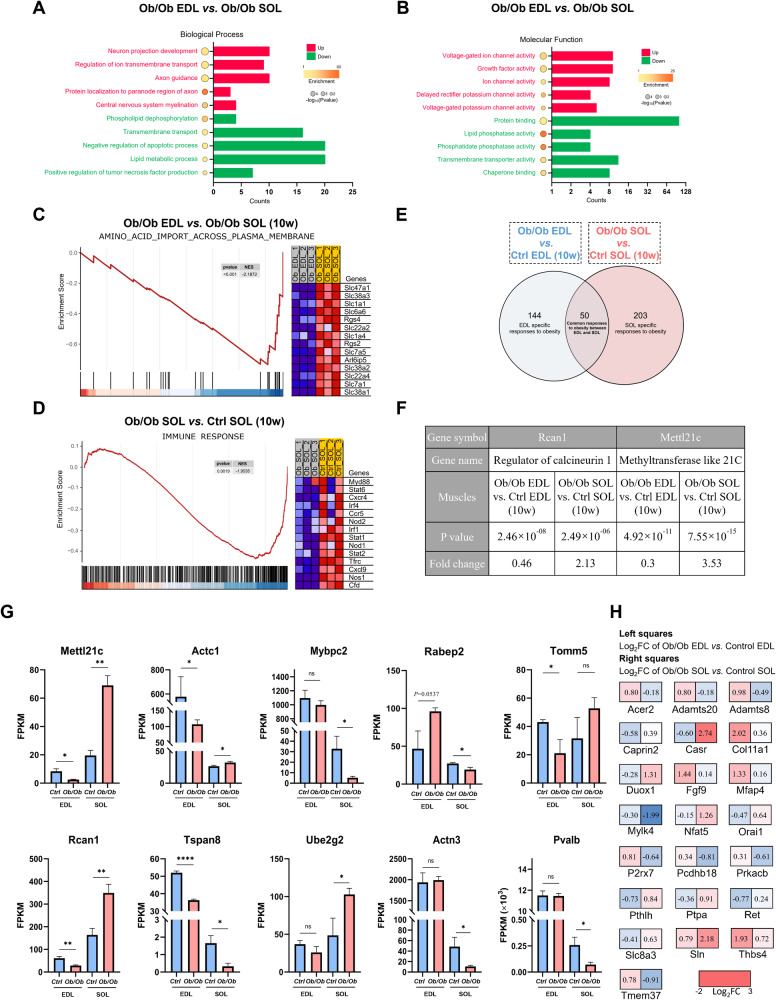
Transcriptomic response of muscle fibers to obesity. **A**, **B** GO analysis showing enriched biological processes and molecular function terms of DEGs between EDL and SOL from Ob/Ob mice. **C** GSEA analysis of amino acid import across plasma membrane. **D** GSEA analysis of immune response. **E** Venn diagram illustrating the genes commonly expressed in EDL and SOL, as well as the DEG specific to EDL and SOL in Ob/Ob mice. **F** Rcan1 and Mettl21c are the only two genes that exhibit opposite expression patterns within the intersection of DEGs in Ob/Ob EDL and Ob/Ob SOL. **G** The top 10 genes exhibiting the most significant expression changes between Ob/Ob EDL and Ob/Ob SOL, with FPKM > 10 in either muscle. **H** Fold change of calcium-related genes in Ob/Ob EDL and Ob/Ob SOL; the left box represents the fold change of relevant genes between control and Ob/Ob EDL, and the right box represents genes in SOL. Data are expressed as the mean ± SEM; *n* = 3; ns indicated not significant, ^*^P < 0.05, ^**^P < 0.01, ^****^P < 0.0001.

To observe the differential response of fast/glycolytic and slow/oxidative fibers to obesity, analysis was conducted on DEGs in SOL and EDL from Ob/Ob mice. After removing 677 fiber-specific DEGs, we conducted a GO analysis on the remaining 417 DEGs. The results revealed significant upregulation of genes related to neuroaxonal processes and ion transport, and a downregulation of lipid metabolic processes in the EDL of obese mice (Fig. 4A, B). Subsequent GSEA analysis of the entire gene set in obese EDL and obese SOL revealed the downregulation of amino acid import in Ob/Ob EDL (Fig. 4C) and the downregulation of immune response in Ob/Ob SOL (Fig. 4D).

The comparison of obese EDL or SOL to their nonobese counterparts revealed 194 DEGs in the EDL muscle between Ob/Ob and control mice, and 253 DEGs in the SOL (Supplementary File 3. Of these, 50 DEGs were common to both groups (Fig. 4E and Supplementary File 3). Out of these 50 DEGs, two genes (Rcan1, Mettl21c) demonstrated opposite expression trends, while the remaining genes exhibited consistent expression patterns (Fig. 4F). After excluding these 50 common DEGs, Table 1 presents the top 10 upregulated and downregulated DEGs in Ob/Ob EDL or SOL relative to their nonobese counterparts. In Ob/Ob EDL, Tnmd, Thbs4, Cilp, Mss51, and Ppp1r1a were specifically upregulated, while Actc1, Amd1, Smox, Amd2, and Cpeb1 were downregulated. Ob/Ob SOL exhibited specific upregulation of Emp1, Tecrl, Sln, Atf3, and Myh3, with Myh4, Mybpc2, Actn3, Ky, and Mylk4 downregulated.

**Table 1:** Top 10 upregulated and downregulated DEGs with an average FPKM > 10 in obese EDL or SOL compared to their nonobese counterparts.

Gene symbol	Gene name	Log_2_FC (Obese_EDL *vs*. WT_EDL)	Regulation	*P* value
Tnmd	Tenomodulin	2.169480478	Up	6.07 × 10^−10^
Thbs4	Thrombospondin 4	1.932935505	Up	2.87 × 10^−23^
Cilp	Cartilage intermediate layer protein, nucleotide pyrophosphohydrolase	1.556304657	Up	1.87 × 10^−08^
Mss51	MSS51 mitochondrial translational activator	1.520067948	Up	0.0001
Ppp1r1a	Protein phosphatase 1, regulatory inhibitor subunit 1 A	1.435062661	Up	5.08 × 10^−13^
Actc1	Actin, alpha, cardiac muscle 1	−2.431256376	Down	1.37 × 10^−05^
Amd1	S-adenosylmethionine decarboxylase 1	−1.968869587	Down	1.28 × 10^−36^
Smox	Spermine oxidase	−1.952857639	Down	4.64 × 10^−26^
Amd2	S-adenosylmethionine decarboxylase 2	−1.565920011	Down	3.22 × 10^−43^
Cpeb1	Cytoplasmic polyadenylation element binding protein 1	−1.443776518	Down	1.98 × 10^−06^

Gene symbol	Gene name	Log_2_FC (Obese_SOL *vs*. WT_SOL)	Regulation	*P* value
Emp1	Epithelial membrane protein 1	2.875011824	Up	0.009
Tecrl	Trans-2,3-enoyl-CoA reductase-like	2.272945128	Up	1.37 × 10^−18^
Sln	Sarcolipin	2.180105104	Up	8.13 × 10^−25^
Atf3	Activating transcription factor 3	2.068130876	Up	0.045
Myh3	Myosin heavy chain 3	1.968983469	Up	3.03 × 10^−19^
Myh4	Myosin heavy chain 4	−3.304873707	Down	0.02
Mybpc2	Myosin binding protein C, fast-type	−2.67345365	Down	0.009
Actn3	Actinin alpha 3	−2.180084625	Down	0.019
Ky	Kyphoscoliosis peptidase	−2.112252104	Down	0.043
Mylk4	Myosin light chain kinase family, member 4	−1.992926495	Down	0.018

Fifty commonly expressed DEGs were excluded from this table.

Genes with an average FPKM > 10 in either sample were considered, including wild-type EDL, wild-type SOL, obese EDL, and obese SOL.

Additionally, genes with distinct expression patterns between EDL and SOL in response to obesity. Figure 4G shows the top 10 genes (FPKM > 10) with the most significant differences. These genes include Mettl21c, Actc1, Mybpc2, Rabep2, Tomm5, Rcan1, Tspan8, Ube2g2, Actn3, and Pvalb. Among them, Mettl21c, Rcan1, Actn3, and Pvalb were associated with calcium homeostasis. Without FPKM limits, numerous calcium signaling factors were found, showing divergent expression trends between Ob/Ob SOL and EDL (Fig. 4H).

To better mimic the physiological state of obesity, diet-induced obesity (DIO) mice were generated by feeding them a 60% high-fat diet starting at 6 weeks old, which continued for 18 weeks (Supplementary Fig. 7A). Immunofluorescence staining of TA muscles in DIO mice revealed similar trends in type I, type IIA, and type IIB muscle fibers as observed in Ob/Ob mice (Supplementary Fig. 7B–C). RNA-Seq and PCA analysis of the glycolytic muscle EDL and oxidative muscle SOL in DIO mice showed comparable expression profiles to those of EDL and SOL in Ob/Ob mice at the overall level (Supplementary Fig. 8A). The muscle atrophy gene marker Fbxo32 was significantly upregulated in the EDL of DIO mice, while no significant changes were observed in SOL (Supplementary Fig. 8B). Two key genes, Mettl21c and Rcan1, identified in Ob/Ob mice were validated in DIO mice (Supplementary Fig. 8C). GSEA results revealed metabolic dysregulation in both the EDL and SOL muscles of DIO mice and Ob/Ob mice. Specifically, glycolytic muscle EDL exhibited a relative increase in oxidative phosphorylation levels, while oxidative muscle SOL showed a relative decrease in these levels (Supplementary Fig. 8D). However, in terms of immunity and inflammation, both glycolytic and oxidative muscle fibers in DIO mice showed downregulation of immune-related signals (Supplementary Fig. 8E).

### Transcriptome changes of fast/glycolytic and slow/oxidative fibers during aging

**Fig. 5:**
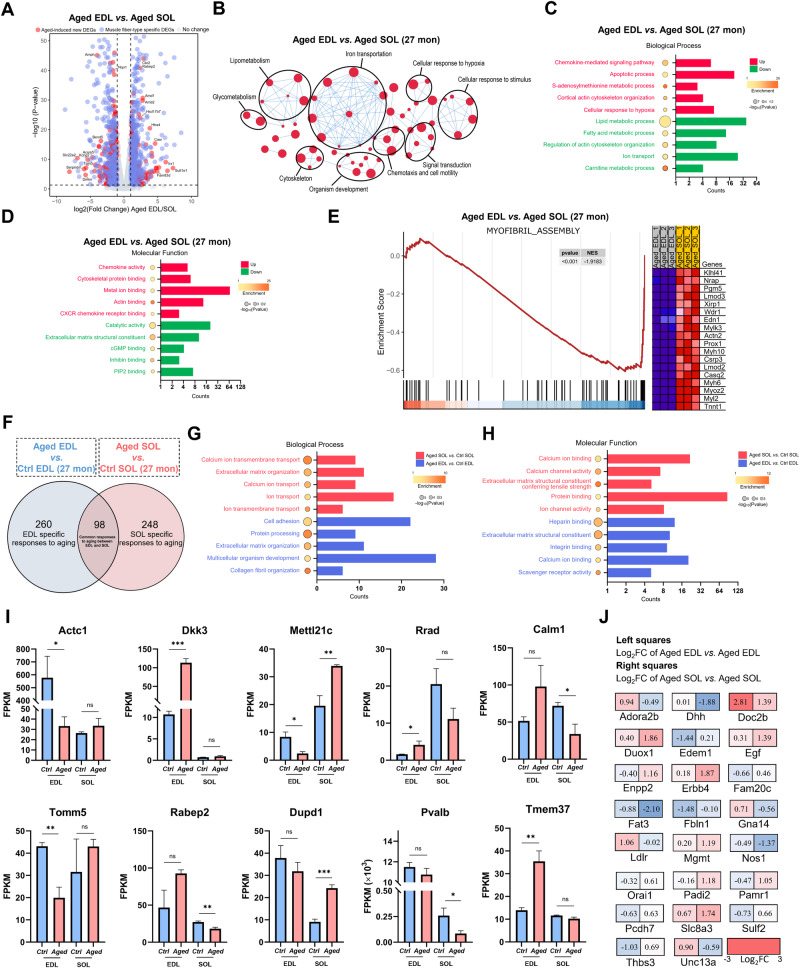
Transcriptomic response of muscle fibers to aging. **A** Volcano plot of DEGs between aged EDL and SOL. **B** Gene-concept network of GO enrichment results. **C**, **D** GO analysis showing enriched biological processes and molecular function terms of DEGs between EDL and SOL from aged mice. **E** GSEA analysis of negative regulation of myofibril assembly in aged EDL. **F** Venn diagram illustrating the genes commonly expressed in aged EDL and SOL, as well as the DEG specific to EDL and SOL in aged mice. **G**, **H** GO analysis showing enriched biological processes and molecular function terms of DEGs specific to aged EDL or aged SOL. **I** The top 10 genes exhibiting the most significant expression changes between EDL and SOL during aging, with FPKM > 10 in either muscle. **J** Fold change of calcium-related genes in EDL and SOL during aging; the left box represents the fold change of relevant genes between control and aged EDL, and the right box represents genes in SOL. Data are expressed as the mean ± SEM; *n* = 3; ns indicated not significant, ^*^P < 0.05, ^**^P < 0.01, ^***^P < 0.001.

To explore the distinct aging responses of fast/glycolytic and slow/oxidative fibers, we analyzed DEGs in aged SOL and EDL. Initially, 1377 protein-coding DEGs were identified. Subsequently, we excluded the 677 muscle fiber-type specific DEGs and further analyzed the remaining 700 protein-coding DEGs to investigate age-specific changes (Fig. 5A and Supplementary File 4).

Based on the visualization of the Gene-Concept network, we observed GO term enrichment predominantly related to iron transport, lipid metabolism, glycometabolism, chemotaxis, cell motility, signal transduction, cellular response to stimulus, and cellular response to hypoxia (Fig. 5B). In the biological process (BP) and molecular function (MF) categories, aged EDL exhibited upregulated genes associated with the chemokine-mediated signaling pathway and apoptosis, along with downregulated genes related to fatty acid metabolism compared to aged SOL (Fig. 5C, D). These findings suggest that fast/glycolytic fibers are more susceptible to inflammation and apoptosis during aging, potentially explaining the rapid atrophy of fast/glycolytic fibers in aging. Additionally, GSEA analysis revealed the negative regulation of myofibril assembly in aged EDL (Fig. 5E).

Furthermore, we compared the DEGs between young and aged fibers. With aging, there are 358 DEGs identified in aged EDL compared to young EDL, and 346 DEGs identified in aged SOL compared to young SOL (Supplementary File 5). Among them, 98 DEGs were common to both SOL and EDL, displaying consistent expression trends (Fig. 5F and Supplementary File 5). These genes likely represent a shared aging response in various fiber types. In addition to these 98 commonly expressed DEGs, the remaining genes are either specific to aged EDL or specific to aged SOL. After excluding these 98 common DEGs, Table 2 presents the top 10 upregulated and downregulated DEGs with an average FPKM > 10 in either muscle samples. In aged EDL, Dkk3, Depp1, Chac1, Mib1, and Fam134b were specifically upregulated, while Actc1, Mybph, Fmod, Slc38a4, and Eif4e were downregulated. Conversely, aged SOL exhibited specific upregulation of Myh3, Nqo1, Dkk2, Cyfip2, and Serpine1, while Tfrc, Gmnn, Clu, Col6a2, and Aqp7 were downregulated. Furthermore, GO analysis of aged EDL or SOL DEGs revealed the extensive involvement of calcium signaling in age-specific responses (Fig. 5G, H).

**Table 2:** Top 10 upregulated and downregulated DEGs with an average FPKM > 10 in aging EDL or SOL compared to their young counterparts.

Gene symbol	Gene name	Log_2_FC (Aged_EDL *vs*. WT_EDL)	Regulation	*P* value
Dkk3	Dickkopf WNT signaling pathway inhibitor 3	3.402647434	Up	1.33 × 10^−87^
Depp1	DEPP1 autophagy regulator	1.978231354	Up	1.64 × 10^−08^
Chac1	ChaC, cation transport regulator 1	1.936698004	Up	0.013
Mib1	MIB E3 ubiquitin protein ligase 1	1.913180379	Up	0.009
Fam134b	Reticulophagy regulator 1	1.674263488	Up	3.03 × 10^−06^
Actc1	Actin, alpha, cardiac muscle 1	−4.117288784	Down	2.68 × 10^−10^
Mybph	Myosin binding protein H	−2.215380324	Down	0.005
Fmod	Fibromodulin	−1.787429702	Down	0.047
Slc38a4	Solute carrier family 38 member 4	−1.567975323	Down	1.76 × 10^−08^
Eif4e	Eukaryotic translation initiation factor 4E	−1.486826431	Down	0.001

Gene symbol	Gene name	Log_2_FC (Aged_SOL *vs*. WT_SOL)	Regulation	*P* value
Myh3	Myosin heavy chain 3	2.351895152	Up	3.43 × 10^−47^
Nqo1	NAD(P)H quinone dehydrogenase 1	1.914230606	Up	1.06 × 10^−24^
Dkk2	Dickkopf WNT signaling pathway inhibitor 2	1.774962666	Up	2.70 × 10^−05^
Cyfip2	Cytoplasmic FMR1 interacting protein 2	1.753302673	Up	2.81 × 10^−26^
Serpine1	Serine (or cysteine) peptidase inhibitor, clade E, member 1	1.738082375	Up	7.92 × 10^−14^
Tfrc	Transferrin receptor	−2.749256582	Down	2.14 × 10^−05^
Gmnn	Geminin	−1.257822209	Down	0.0003
Clu	Clusterin	−1.254860483	Down	9.80 × 10^−06^
Col6a2	Collagen, type VI, alpha 2	−1.241430598	Down	0.006
Aqp7	Aquaporin 7	−1.180129885	Down	0.0003

Ninety-eight commonly expressed DEGs were excluded from this table.

Genes with an average FPKM > 10 in either sample were considered, including wild-type EDL, wild-type SOL, aged EDL, and aged SOL.

Comparing the expression pattern between aged SOL and EDL muscles, Fig. 5I displays the top 10 genes (Actc1, Dkk3, Mettl21c, Rrad, Calm1, Tomm5, Rabep2, Dupd1, Pvalb, Tmem37) with an average FPKM > 10 in either muscle, showing the most significant expression differences between the two muscle types. Notably, five (Mettl21c, Rrad, Calm1, Pvalb, and Tmem37) out of ten genes were actively involving in calcium-related pathway or calcium homeostasis. When considering all genes without FPKM restrictions, a greater number of calcium-related genes were identified (Fig. 5J).

To explore the mechanistic insight into the difference between aged EDL and aged SOL, transcriptional regulatory analyses were conducted using TRRUST database [26]. Our analysis revealed that aged EDL in mice exhibited a pronounced inflammatory phenotype, with NF-KB acting as one of the central transcription factors, a feature not seen in aged SOL (Supplementary Fig. 9A). Furthermore, the analysis of secretory factors revealed minimal overlap between aged EDL and SOL, with aged EDL displaying increased expression of various pro-atrophy secretory factors, including Dkk3 and inflammatory cytokines such as Ccl8, Cxcl10, and Cxcl13 (Supplementary Fig. 9B, C). Supplementary Fig. 9D lists the top 5 upregulated inflammatory factors in aged EDL.

### Interventions targeting calcium ion transport increase the proportion of slow/oxidative fibers

**Fig. 6:**
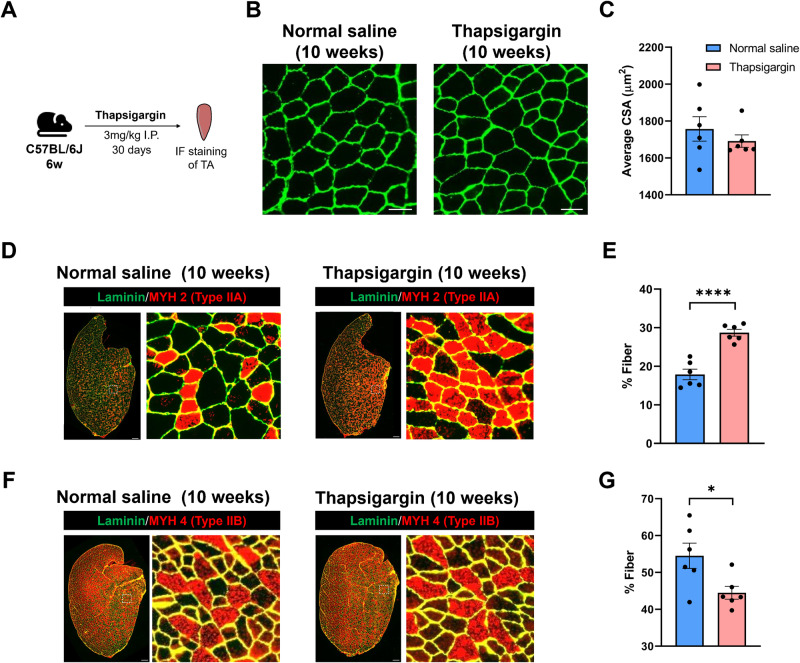
Administration of thapsigargin significantly increases the number of oxidative fibers in TA. **A** Mice were injected with thapsigargin (3 mg/kg) or normal saline intraperitoneally (i.p.) every day for 1 months. **B** Immunofluorescence staining of laminin for TA sections, Scale bar: 50 μm. **C** Statistical results of the average CSA of TA fibers in mice treated with or without thapsigargin. **D** Immunofluorescence staining of MYH2 of TA in control, aged, and Ob/Ob mice. Scale bar: 200 μm. **E** Statistical results of the type IIA fibers in TA. **F** Immunofluorescence staining of MYH4 of TA, Scale bar: 200 μm. **G** Statistical results of the type IIB fibers in TA. Data are expressed as the mean ± SEM; *n* = 6; ^*^P < 0.05, ^****^P < 0.0001.

Due to the extensive involvement of calcium-related signaling in the response of fast and slow muscles to aging and obesity, we investigated whether changes in cytoplasmic calcium concentration led to alterations in muscle fiber composition. Thapsigargin, a non-competitive inhibitor of the sarco/endoplasmic reticulum Ca^2+^-ATPase (SERCA), was administered via 30-day intraperitoneal injection (3 mg/kg) to 6-week-old WT mice. After 30 days, TA muscles were collected and subjected to immunofluorescence staining of MYH2 and MYH4 (Fig. 6A). Thapsigargin injection resulted in a significant increase in the proportion of oxidative fibers while having no impact on CSA (Fig. 6B–G). Additionally, one month of thapsigargin injections had no adverse effects on mouse body weight, muscle mass, muscle function, or cardiac function (Supplementary Fig. 10).

## Discussion

Research on muscle fiber type changes in aging and obesity is limited and inconsistent. Our study explores transcriptome differences between EDL (rich in glycolytic fibers) and SOL (rich in oxidative fibers) during aging and obese conditions, revealing type-specific alterations. We uncovered that in muscles dominated by oxidative fibers (such as SOL), the proportion of oxidative fibers remain unchanged during aging and obesity. In contrast, muscles dominated by glycolytic fibers (such as TA, quadriceps, and EDL) experience a significant decrease in oxidative fibers. Mechanism analysis showed that during aging and obesity, fast/glycolytic fibers tend to express genes associated with muscle atrophy and inflammation, while slow/oxidative fibers tend to express genes involved in cellular protection. Besides, significant differences in calcium-related pathways were observed during aging and obesity. Treatment with a calcium channel inhibitor significantly increased the number of oxidative fibers. These findings shed light on the unique changes occurring in different fiber types and their potential implications in the context of aging and obesity.

Our findings provide new explanations for existing researches. Since the majority of current human studies are based on the vastus lateralis muscle, which is primarily composed of slow/oxidative muscle fibers (with fast/glycolytic fibers constituting only 10–20%) [27–29], the upregulation of cellular protective genes in slow/oxidative fibers may lead to the maintenance or even increase in the content of slow/oxidative fibers in this muscle with aging. In contrast, the quadriceps muscle in mice is predominantly composed of fast/glycolytic fibers, and the secretion of atrophy and inflammatory signals from these fibers contributes to the decline in slow/oxidative fiber content observed in the quadriceps of aging and obese mice. One exception is that many studies suggest a decrease in slow/oxidative muscle fibers in the vastus lateralis muscle of obese humans [30]. We speculate that this discrepancy may be attributed to the presence of intramuscular and intermuscular fat, as evidence suggests that the inflammatory factors secreted by intramuscular and intermuscular fat are more abundant than those from visceral and subcutaneous fat [31]. Consequently, these fat tissues could substitute the role of fast/glycolytic fibers, resulting in a reduction of slow/oxidative muscle fibers in the vastus lateralis muscle of obese humans. Our theory may also help to explain the inconsistency in the fiber type composition in muscles from different anatomical locations, as observed in different studies [10, 11, 32]. Similar to our study, Sheard et al. examined muscle fiber composition in EDL and SOL, and found that in aging male mice, the proportion of type I fibers in EDL decreased from 10% at 6 months to 0.5% at 24 months. Conversely, in SOL, the proportion of type I fibers increased from 30 to 38% [33]. Crupi et al. reported that fibers expressing type I myosin were observed in the tibialis anterior muscles of young mice, although in limited numbers, and were frequently entirely absent in aged TA muscles [13].

Our study revealed that fast/glycolytic fibers exhibited an inflammatory phenotype, with NF-κB acting as one of the central transcription factors. Besides, fast/glycolytic fibers tend to upregulate genes such as Dkk3, Ccl8, Cxcl10, Cxcl13, Fbxo32, Depp1, and Chac1 during the aging and obese process, which is known to promote muscle atrophy [34, 35]. Our study is consistent with previous studies that fast/glycolytic fibers exhibit higher expression of Atrogin-1 and MuRF proteins than slow/oxidative fibers during immobilization [36]. In contrast, slow/oxidative fibers exhibit specific upregulation of Nqo-1 and downregulation of Tfrc during aging. Due to the frequently observed iron overload during aging [37], the downregulation of Tfrc and upregulation of the antioxidant protein Nqo-1 play a significant role in protecting oxidative stress.

In our study, we found that Mettl21c is significantly upregulated in slow/oxidative fibers, but is significantly downregulated in the fast/glycolytic fibers during both aging and obesity process. This expression pattern of Mettl21c highlights its specific and unique role in muscle fibers. A previous study showed that Mettl21c is exclusively expressed in slow MYH7-positive muscle fibers, and deletion of Mettl21c has been associated with decreased running performance and the notable accumulation of autophagic vacuoles [38]. Besides, previous studies have suggested a relationship between Mettl21c and calcium signaling [39], and our research revealed significant differences in calcium-related genes between fast/glycolytic and slow/oxidative fibers during aging and obesity. Despite of that, further investigations are needed to elucidate whether Mettl21c influences fiber composition through calcium homeostasis.

In conclusion, fast/glycolytic and slow/oxidative fibers exhibit distinct responses to aging and obesity. Fast/glycolytic fibers tend to express pro-atrophy genes, whereas slow/oxidative fibers upregulate cytoprotective genes and downregulate genes that may mediate cell damage during aging and obesity. Additionally, we observed significant differences in the expression of calcium-related genes in response to aging and obesity between fast/glycolytic and slow/oxidative fibers. Treatment with a calcium channel inhibitor significantly increased the quantity of oxidative fibers. Our study provides supporting evidence for transcriptomic differences in fiber types under pathophysiological conditions and lays the theoretical foundation for modulating muscle fiber types in the treatment of diseases.

### Supplementary information

Supplementary materials

Supplementary Files

## Data Availability

The RNA-seq data have been deposited in the NCBI SRA database under accession codes PRJNA1009685 and PRJNA1108514. Data in this study is available upon reasonable request from the corresponding author.
