# Supplementary material for: Transcriptome profiling of fast/glycolytic and slow/oxidative muscle fibers in aging and obesity
Source: Cell Death Dis. 2024 Jun 28;15(6):459. doi: 10.1038/s41419-024-06851-y (PMC11213941; doi:10.1038/s41419-024-06851-y)
Supplement: Supplementary file 1 — Supplementary materials [file 41419_2024_6851_MOESM1_ESM.pdf]

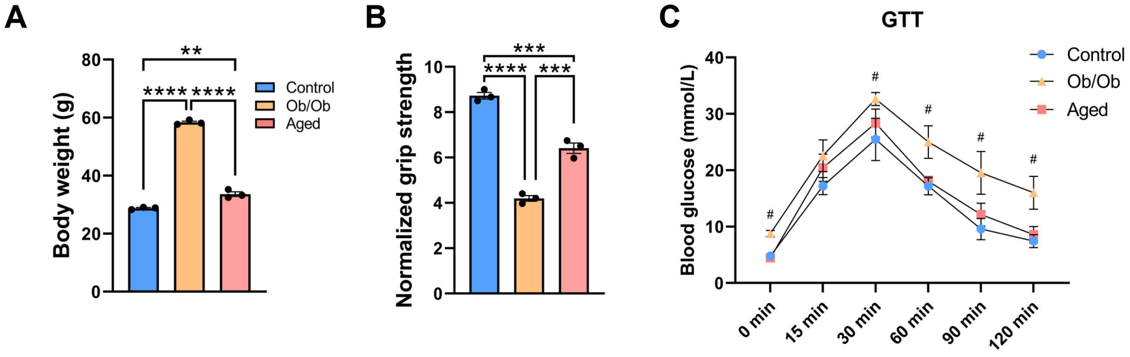

Supplementary Figure 1. Phenotype of control (10 weeks), Ob/Ob (10 weeks), and aged (27 months) C57BL/6 mice. **(A)** Body weight of control (10 weeks), Ob/Ob (10 weeks), and aged (27 months) mice. **(B)** Grip strength, data were normalized by body weight. **(C)** Glucose tolerance test (GTT). n=3-6; ** indicated *P*<0.01, *** indicated *P*<0.001, **** indicated *P*<0.0001; $^{\#}$ indicated control group *vs.* Ob/Ob group, *P*<0.05.

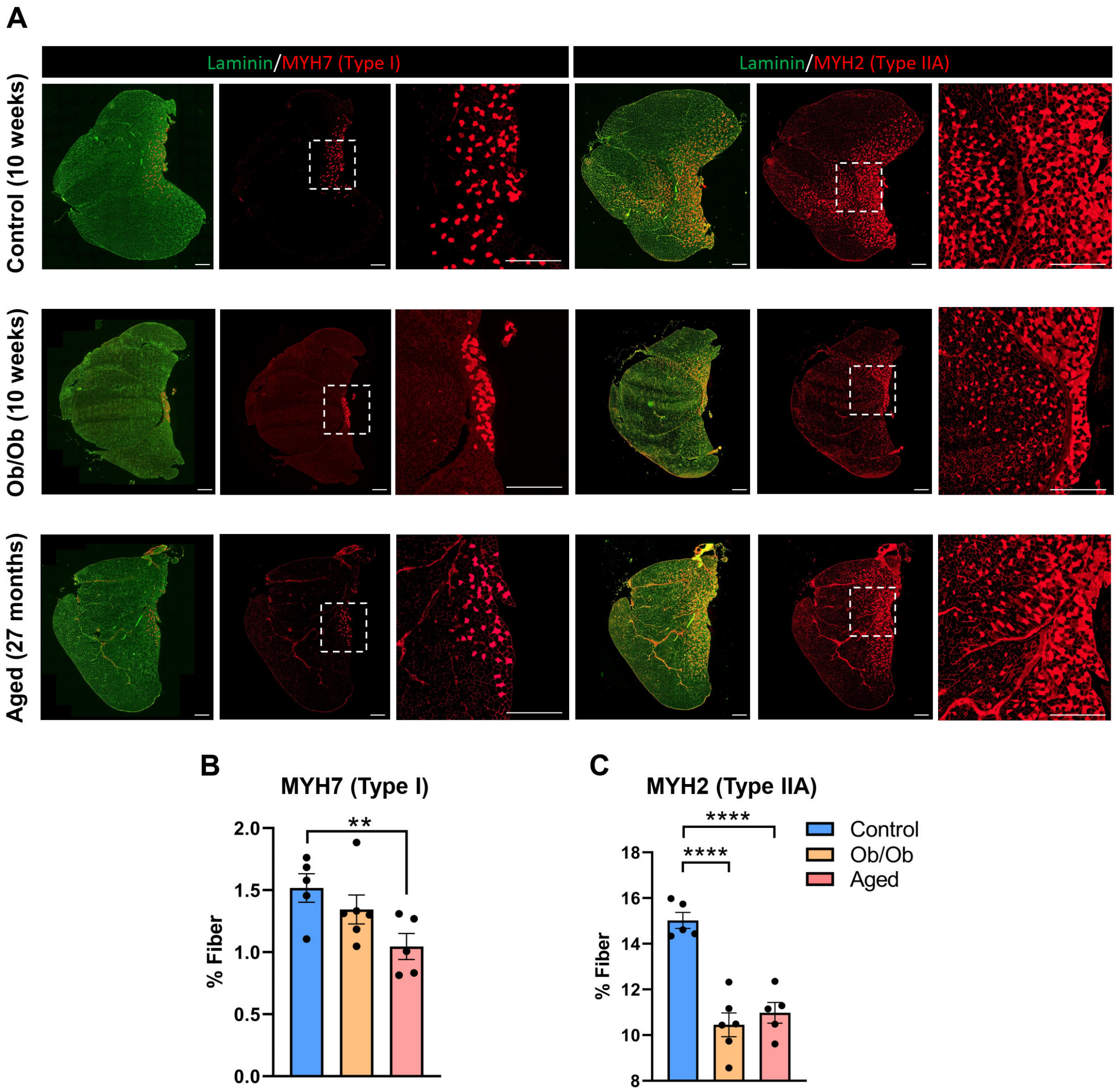

Supplementary Figure 2. Aging and obesity lead to a decrease in the proportion of oxidative fibers in quadriceps. **(A)** Immunofluorescence staining of type I and IIA fibers in control, aged, and Ob/Ob mice. **(B)** Statistical results of the proportion of type I fibers. **(C)** Statistical results of the proportion of type IIA fibers. n=5-6, ** indicated P<0.01, **** indicated P<0.0001.

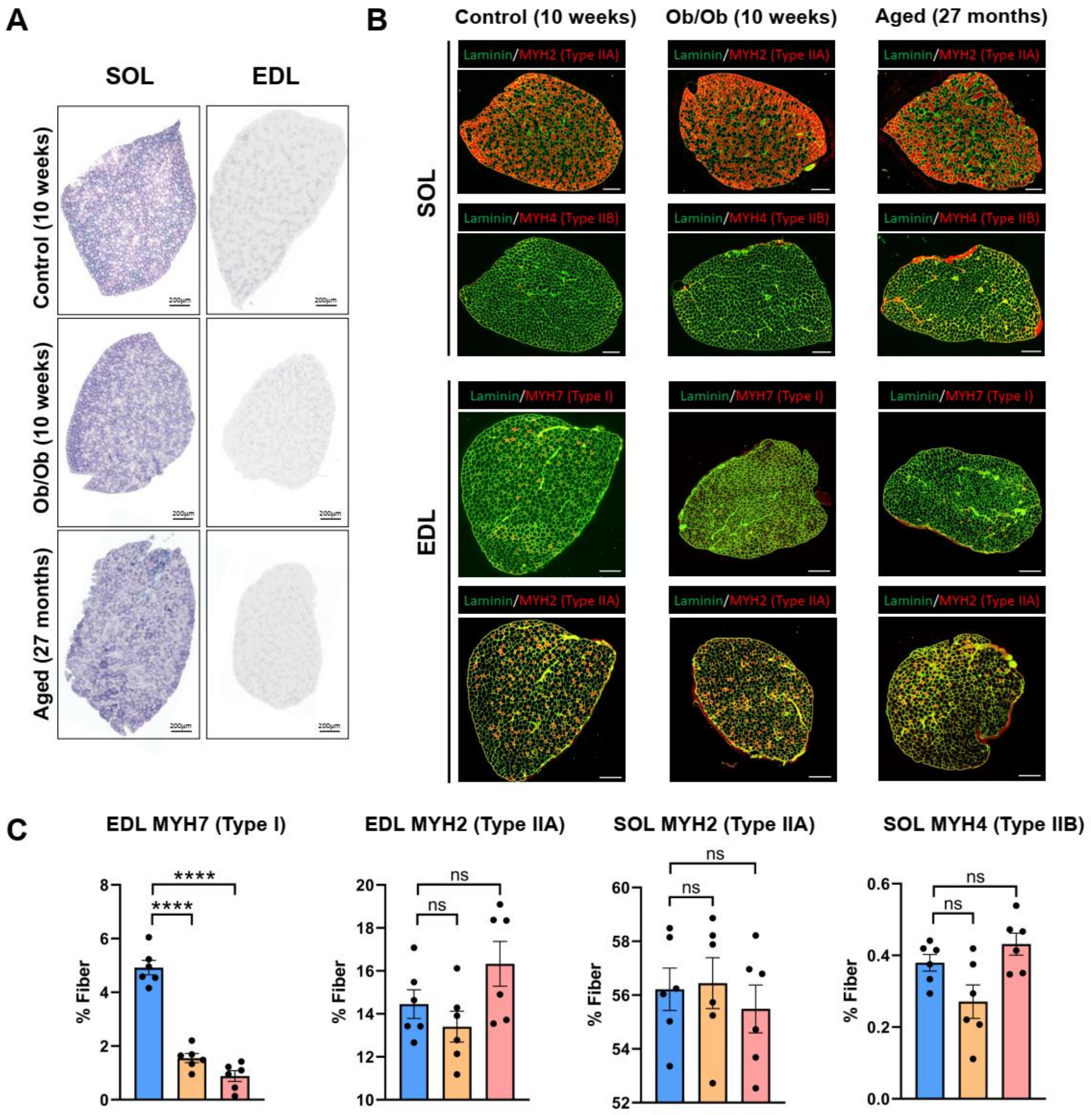

Supplementary Figure 3. SOL predominantly consists of oxidative muscle fibers, while EDL is primarily composed of glycolytic fibers. **(A)** SDH staining of TA sections in control, aged, and Ob/Ob mice. **(B)** Immunofluorescence staining of type IIA and IIB for SOL, and type I and IIA for EDL. **(C)** Statistical results of the proportion of type I, IIA, and IIB fibers in EDL or SOL. n=6, **** indicated P<0.0001.

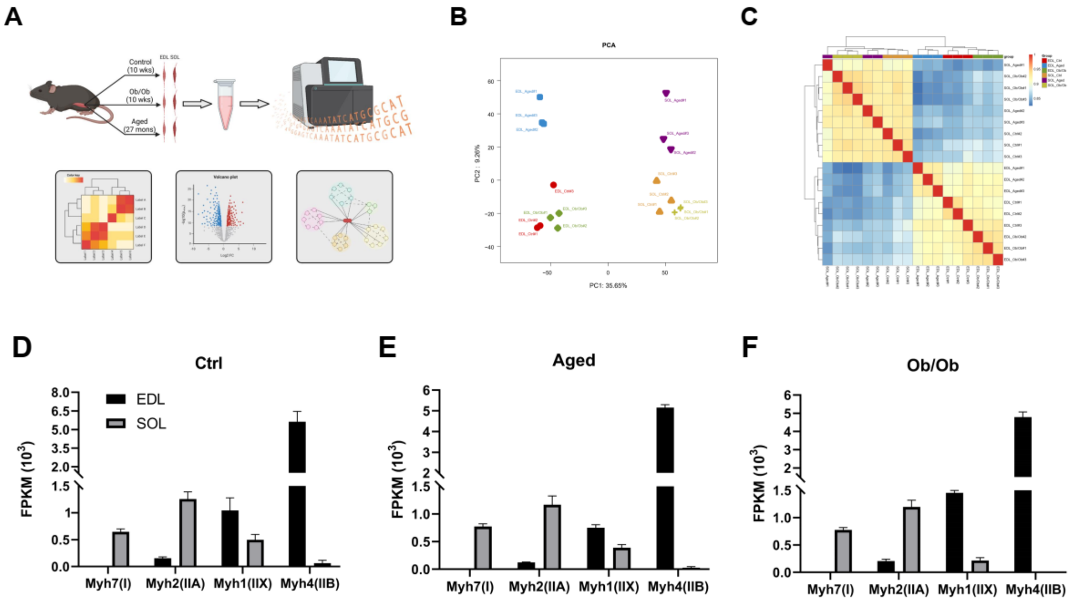

Supplementary Figure 4. Schematic diagram of RNA sequencing. **(A)** Scheme representing the mice model, experimental set-up and assays employed. Figure is created by Biorender. **(B-C)** Principal component analysis (PCA) and correlation heatmap illustrating the similarity between transcriptomic profiles generated from RNA-Seq samples. **(D-F)** Expression level of Myh1, Myh2, Myh4, and Myh7 in EDL and SOL from control, aged, and Ob/Ob mice analyzed by RNA-seq. n=3.

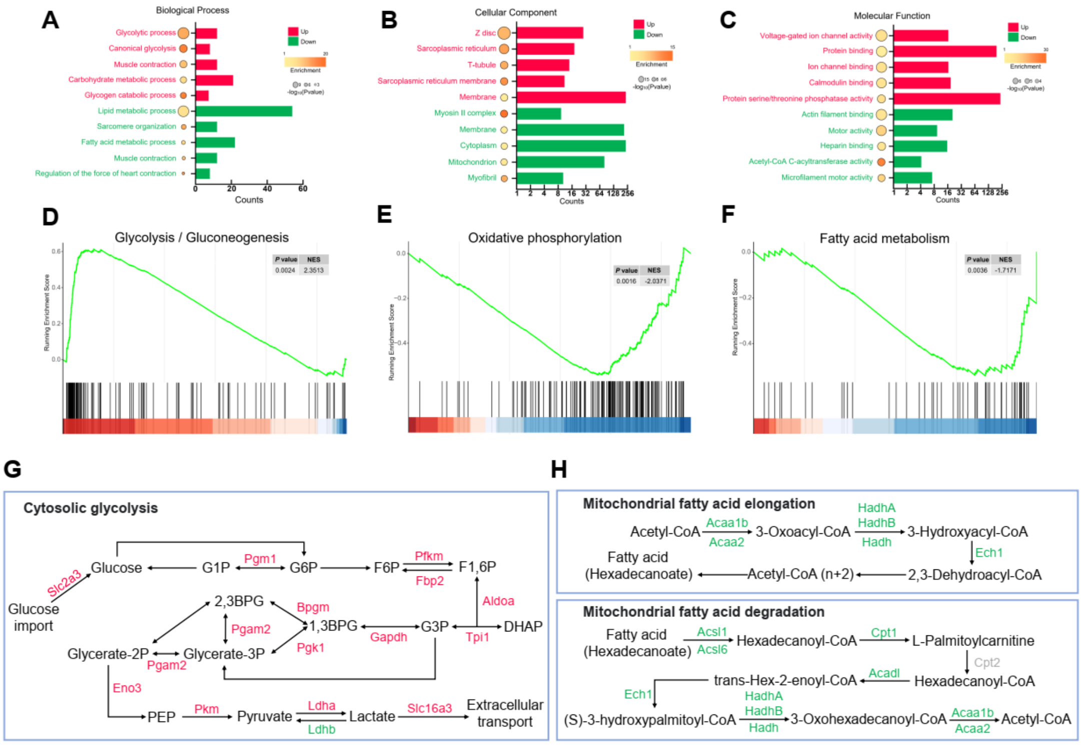

Supplementary Figure 5. RNA-Seq analysis of DEGs between EDL and SOL from control mice. **(A-C)** GO analysis showing enriched biological processes, molecular function, and cellular component terms of DEGs between EDL and SOL from control mice. **(D-F)** Representative differences of transcriptome patterns between EDL and SOL generated by GSEA analysis. **(G-H)** DEGs involved in glycolysis and fatty acid metabolism among EDL and SOL.

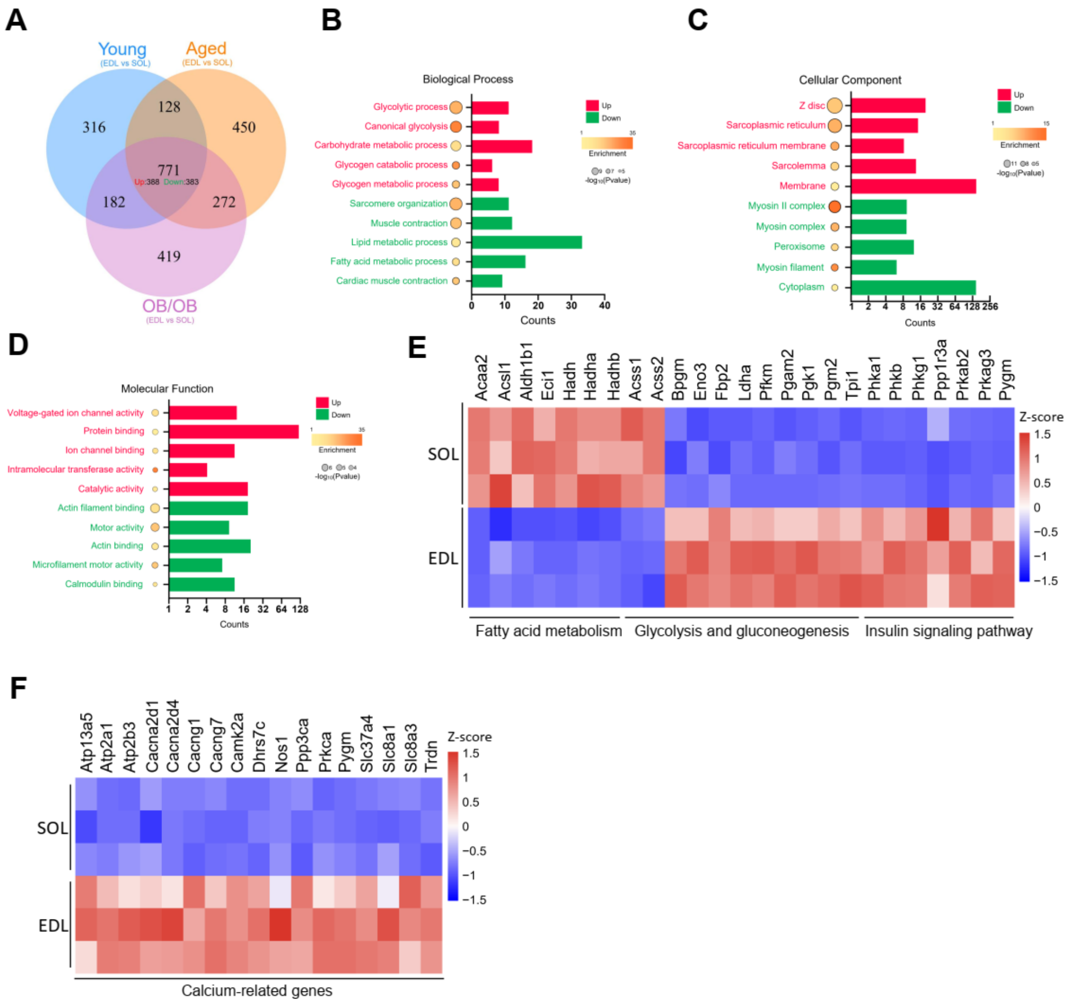

Supplementary Figure 6. Identification of commonly expressed genes with muscle fiber type specificity across different groups. **(A)** The Venn plot demonstrated that there were 771 commonly expressed DEGs (677 protein-coding genes) between EDL and SOL that were shared among control, aged, and Ob/Ob mice. **(B-D)** GO analysis of these commonly expressed protein-coding gene DEGs. **(E-F)** Heatmap of genes involving in fatty acid metabolism, glycolysis and gluconeogenesis, insulin signaling pathway, and calcium homeostasis.

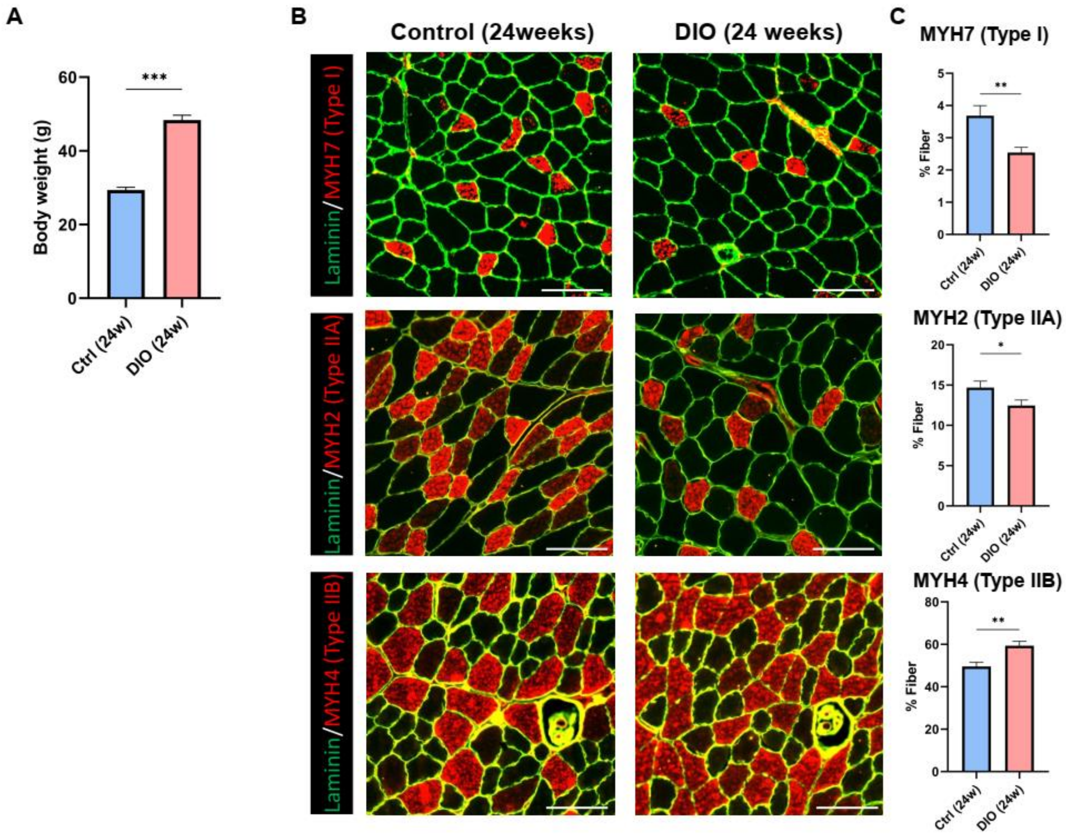

Supplementary Figure 7. Diet-induced obese (DIO) mice exhibited similar muscle fiber type changes in the TA as observed in Ob/Ob mice. **(A)** Body weight of DIO mice and littermate controls. **(B)** Immunofluorescence staining of MYH7, MYH2, MYH4 of TA sections. Scale bar: 100 μm. **(C)** Statistical results of the proportion of type I, IIA, IIB fibers. Data are expressed as percentages; n=3 or 6; * indicated P<0.05, ** indicated P<0.01.

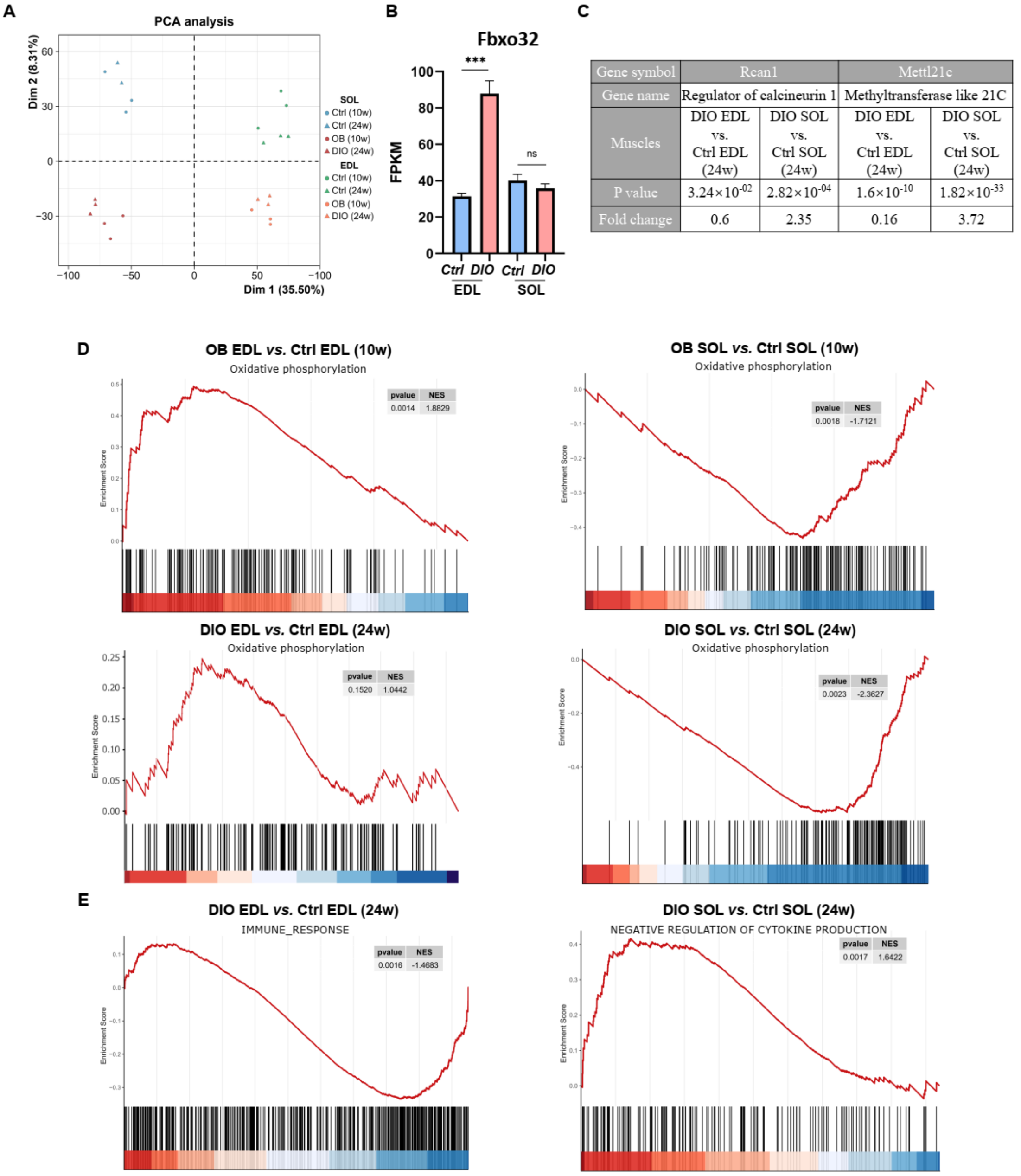

Gene symbol	Rcan1		Mettl21c	
Gene name	Regulator of calcineurin 1		Methyltransferase like 21C	
Muscles	DIO EDL vs. Ctrl EDL (24w)	DIO SOL vs. Ctrl SOL (24w)	DIO EDL vs. Ctrl EDL (24w)	DIO SOL vs. Ctrl SOL (24w)
P value	3.24×10^{-02}	2.82×10^{-04}	1.6×10^{-10}	1.82×10^{-33}
Fold change	0.6	2.35	0.16	3.72

Supplementary Figure 8. DIO mice and Ob/Ob mice had similar overall transcriptome profiles. **(A)** PCA analysis. **(B)** PFKM of Fbxo32 gene in EDL and SOL of DIO and control mice. **(C)** Expression level of Rcan1 and Mettl21c in EDL and SOL of DIO and control mice. **(D)** GSEA analysis of oxidative phosphorylation in DIO and Ob/Ob mice. **(E)** GSEA analysis of immune-related pathways.

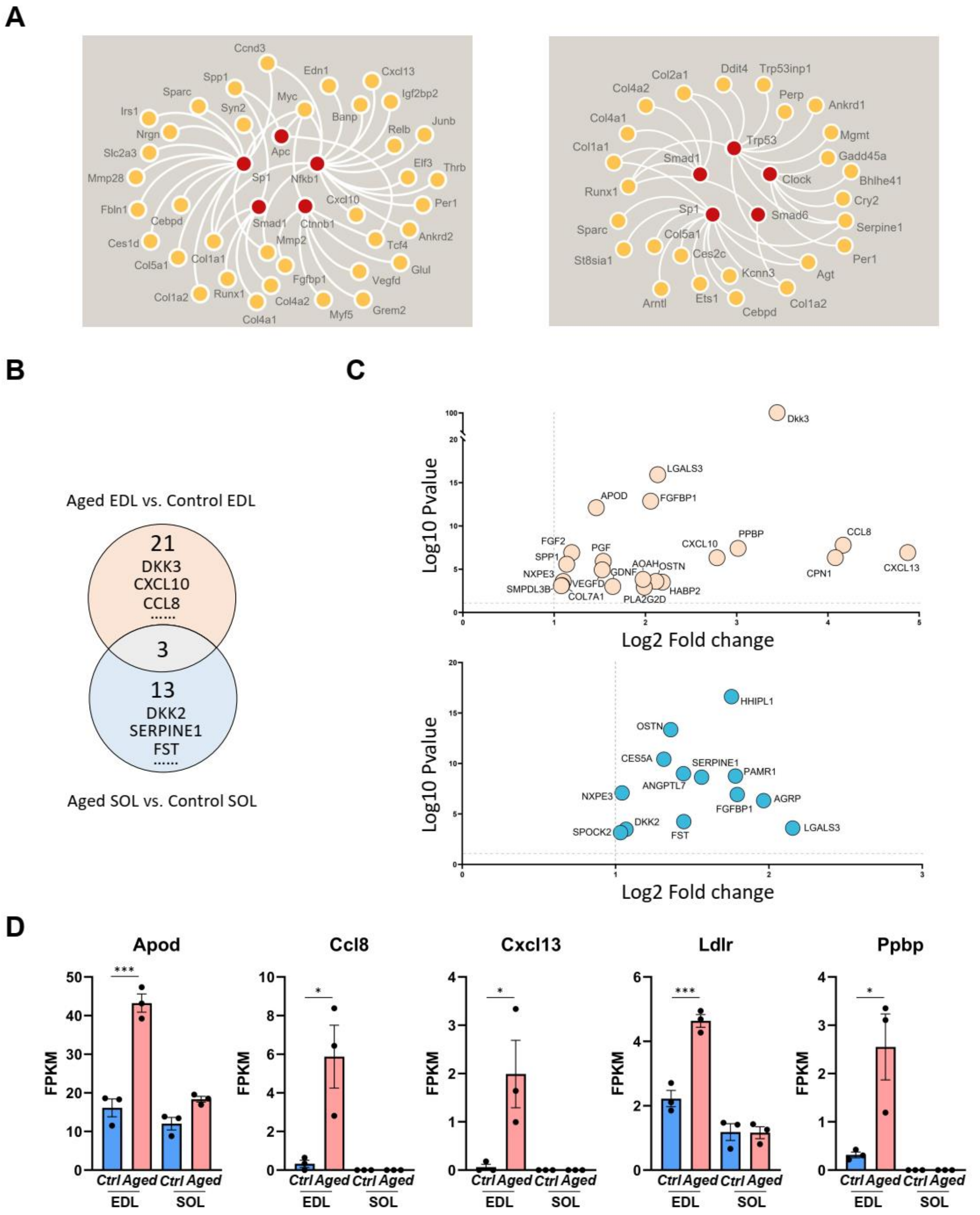

Supplementary Figure 9. Aged EDL exhibited a pronounced inflammatory phenotype. **(A)** Transcriptional regulatory network analysis of DEGs between control and aged EDL, as well as between control and aged SOL. **(B)** Venn diagram illustrating the shared upregulated genes encoding secreted proteins. **(C)** Volcano plot displaying the upregulated genes encoding secreted proteins. **(D)** The top 5 upregulated genes encoding proteins related to inflammation.

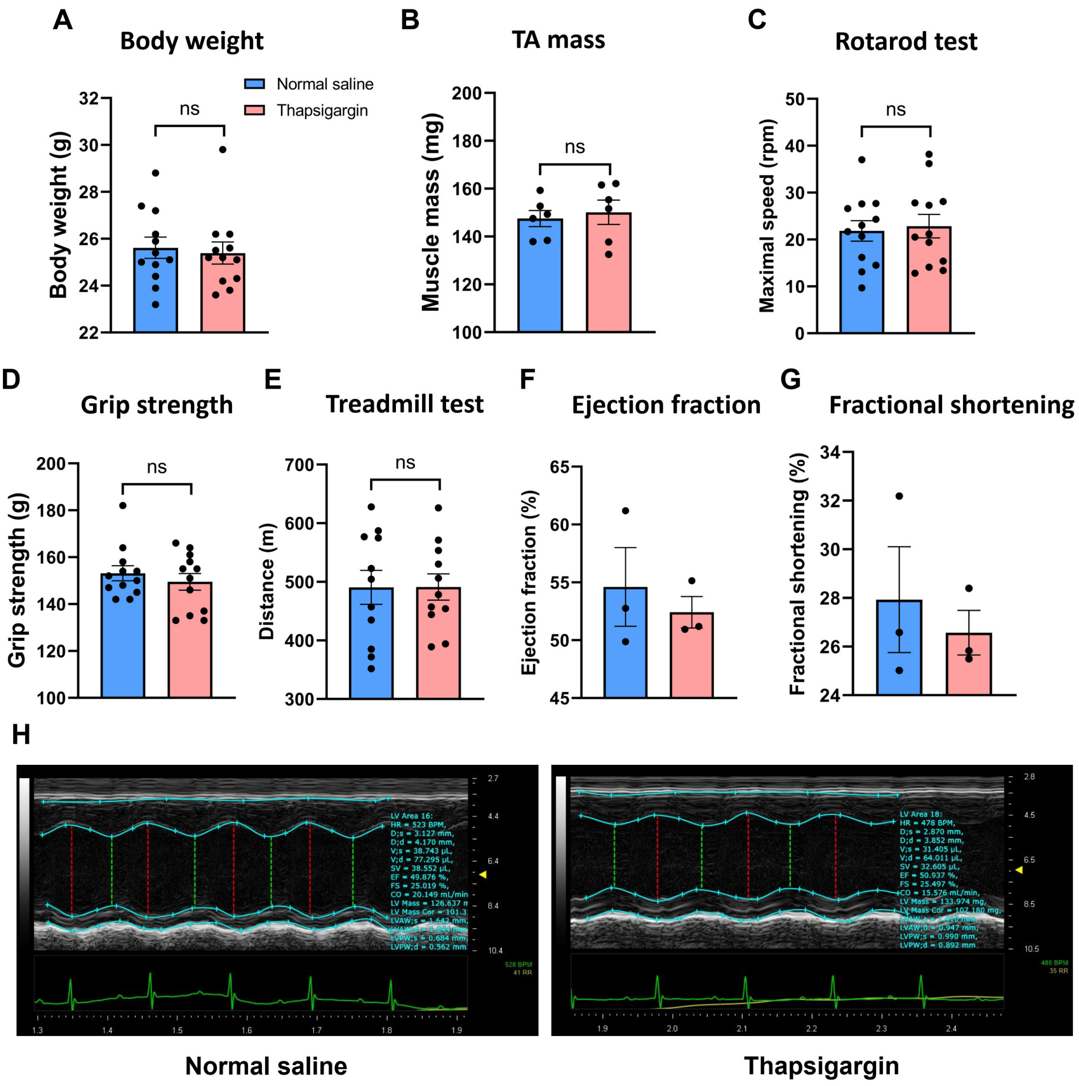

Supplementary Figure 10. One month of thapsigargin injections had no detrimental effects on muscle and cardiac function. **(A)** Body weight. **(B)** TA mass. **(C)** Rotarod test. **(D)** Grip strength test. **(E)** Treadmill test. **(F)** Ejection fraction. **(G)** Fractional shortening. **(H)** Representative images of mouse cardiac ultrasound show no significant difference in cardiac function between the thapsigargin-injected and normal saline groups. n=3 or 12.

Supplementary Table 1. Top 10 upregulated and 10 downregulated DEGs with an average FPKM>10 in EDL or SOL.

Gene symbol	Gene name	Log_2FC (WT_EDL *vs.* WT_SOL)	Regulation	P value
Myh4	Myosin heavy chain 4	6.470873738	Up	4.85×10^{-18}
Mss51	MSS51 mitochondrial translational activator	5.831504388	Up	4.23×10^{-35}
Mstn	Myostatin	5.534096604	Up	7.77×10^{-96}
Pvalb	Parvalbumin	5.475860327	Up	5.41×10^{-70}
Actn3	Actinin alpha 3	5.315657531	Up	1.17×10^{-20}
Mybpc2	myosin binding protein C, fast-type	5.06095736	Up	6.42×10^{-19}
Tspan8	Tetraspanin 8	4.963672006	Up	1.97×10-^{65}
Mybph	Myosin binding protein H	4.507055986	Up	2.61×10^{-09}
Kcnc4	Potassium voltage gated channel, Shaw-related subfamily, member 4	4.48659624	Up	7.49×10^{-126}
Actc1	Actin, alpha, cardiac muscle 1	4.446559479	Up	4.30×10^{-40}
Myl3	Myosin light chain 3	-8.089464945	Down	1.43×10^{-26}
Myh7	Myosin heavy chain 7	-7.527033817	Down	3.34×10^{-10}
Tnni1	Troponin I, skeletal, slow 1	-6.999636907	Down	6.32×10^{-17}
Tnnc1	Troponin C, cardiac/slow skeletal	-6.943041949	Down	1.30×10^{-24}
Strit1	Small transmembrane regulator of ion transport 1	-6.748057324	Down	0.002
Tnnt1	Troponin T1, skeletal, slow	-6.707614629	Down	2.42×10^{-35}
Myl2	myosin, light polypeptide 2, regulatory, cardiac, slow	-6.560322344	Down	2.15×10^{-117}
Atp2a2	ATPase, Ca2+ transporting, cardiac muscle, slow twitch 2	-5.917923961	Down	2.74×10^{-180}
Sim2	Single-minded family bHLH transcription factor 2	-5.189073277	Down	2.38×10^{-45}
Tpm3	Tropomyosin 3	-5.026340947	Down	5.80×10^{-182}

Data was derived from control EDL *vs.* control SOL.
