# Supplementary material for: Transcriptome profiling of fast/glycolytic and slow/oxidative muscle fibers in aging and obesity
Source: Cell Death Dis. 2024 Jun 28;15(6):459. doi: 10.1038/s41419-024-06851-y (PMC11213941; doi:10.1038/s41419-024-06851-y)
Supplement: Supplementary file 2 — Supplementary Files [file 41419_2024_6851_MOESM2_ESM.zip › Supplementary Files/File legend.docx]

**Supplementary Files 1.** Transcriptional differences between fast- and slow-twitch fibers in 10-week-old wild-type mice.

**Supplementary Files 2.** DEGs that consistently appear in the control, aged, and Ob/Ob groups.

**Supplementary Files 3.** DEGs between aged EDL and SOL.

**Supplementary Files 4.** Age-specific genes in EDL and SOL.

**Supplementary Files 5.** Obesity-specific genes in EDL and SOL.
